# Magnetic resonance-guided focused ultrasound thalamotomy in essential tremor subtypes: a phenotype-based insight into gait and balance

**DOI:** 10.1093/braincomms/fcag076

**Published:** 2026-03-11

**Authors:** Isabel Sastre-Bataller, Rebeca Conde-Sardón, Marina Campins-Romeu, Carlos Morata-Martínez, Raquel Baviera-Muñoz, Julia Pérez-García, Mireya Losada-López, María J Ibáñez-Juliá, José L León-Guijarro, Andrés M Lozano, Antonio Gutiérrez-Martín, Irene Martínez-Torres

**Affiliations:** Movement Disorders Unit, Department of Neurology, Hospital La Fe, 46026 Valencia, Spain; Unit of Functional Neurosurgery, Department of Neurosurgery, Hospital La Fe, 46026 Valencia, Spain; Neuroscience Department, ASCIRES Biomedical Group, 46010 Valencia, Spain; Movement Disorders Unit, Department of Neurology, Hospital La Fe, 46026 Valencia, Spain; Movement Disorders Unit, Department of Neurology, Hospital La Fe, 46026 Valencia, Spain; Neuroscience Department, ASCIRES Biomedical Group, 46010 Valencia, Spain; Movement Disorders Unit, Department of Neurology, Hospital La Fe, 46026 Valencia, Spain; Movement Disorders Unit, Department of Neurology, Hospital La Fe, 46026 Valencia, Spain; Neuroscience Department, ASCIRES Biomedical Group, 46010 Valencia, Spain; Neuroscience Department, ASCIRES Biomedical Group, 46010 Valencia, Spain; Neuroscience Department, ASCIRES Biomedical Group, 46010 Valencia, Spain; Neuroscience Department, ASCIRES Biomedical Group, 46010 Valencia, Spain; Division of Neurosurgery, Department of Surgery, Toronto Western Hospital, Toronto, ON, Canada M5T 2S8; Unit of Functional Neurosurgery, Department of Neurosurgery, Hospital La Fe, 46026 Valencia, Spain; Neuroscience Department, ASCIRES Biomedical Group, 46010 Valencia, Spain; Movement Disorders Unit, Department of Neurology, Hospital La Fe, 46026 Valencia, Spain; Neuroscience Department, ASCIRES Biomedical Group, 46010 Valencia, Spain

**Keywords:** focused ultrasound, essential tremor, dystonic tremor, thalamotomy, balance

## Abstract

Magnetic resonance-guided focused ultrasound (MRgFUS) thalamotomy is an established treatment for medication-refractory essential tremor (ET). However, evidence regarding its efficacy and safety in ET with imbalance (ET-I) and dystonic tremor (DT) remains limited, particularly with respect to gait outcomes. We prospectively evaluated whether tremor control and gait stability after unilateral MRgFUS ventral intermediate nucleus (VIM) thalamotomy differed according to tremor phenotype and baseline gait status. Eighty-one patients were enrolled (40 ET, 11 ET-I, 30 DT; 59.3% male; median age 73 years) and assessed with the clinical rating scale for tremor, Berg balance scale, and tandem gait performance at baseline, 6 months and 12 months. Phenotypes were assigned by two movement disorder specialists with substantial inter-rater agreement (κ & 0.72). Seventy-six patients achieved at least one effective sonication (≥55°C) and were included in outcome analyses. At 6 months, treated hand tremor score improved by 80% in ET, 70% in ET-I, and 71% in DT. At 12 months, a sustained improvement of 50% or greater was maintained in 94% of ET patients, 71% of ET-I patients, and 82% of DT patients. While ET patients maintained stable tremor benefit at 12 months (78% improvement), ET-I and DT showed a modest decline (63% and 63%, respectively). Disability scores followed similar trajectories. Tandem gait remained stable in 87% of patients at 12 months, with gait worsening being infrequent and generally mild (15.7% at 6 months; 13.3% at 12 months) and not associated with phenotype, baseline instability or age. Notably, some ET-I and DT patients improved from moderate to mild impairment, and only two patients developed severe gait disturbances at 12 months. Adverse events, systematically assessed at 1, 6 and 12 months, were mild and transient, and no DT patient exhibited worsening of dystonia. These findings indicate that MRgFUS VIM thalamotomy is a relatively safe and effective option across tremor phenotypes, including patients with mild baseline imbalance, with most individuals achieving durable tremor benefit and preserved gait function. Our results support phenotype-based patient selection and reinforce the value of long-term, individualized follow-up, particularly in older or clinically complex cases.

## Teaser

Sastre-Bataller *et al*. reported that high-intensity focused ultrasound thalamotomy provides sustained tremor control across essential tremor (ET) phenotypes with minimal impact on gait and balance. These results support its use in ET with mild imbalance and dystonic tremor and underscore the importance of phenotype-adapted long-term follow-up.

## Graphical Abstract

**Graphical Abstract:**
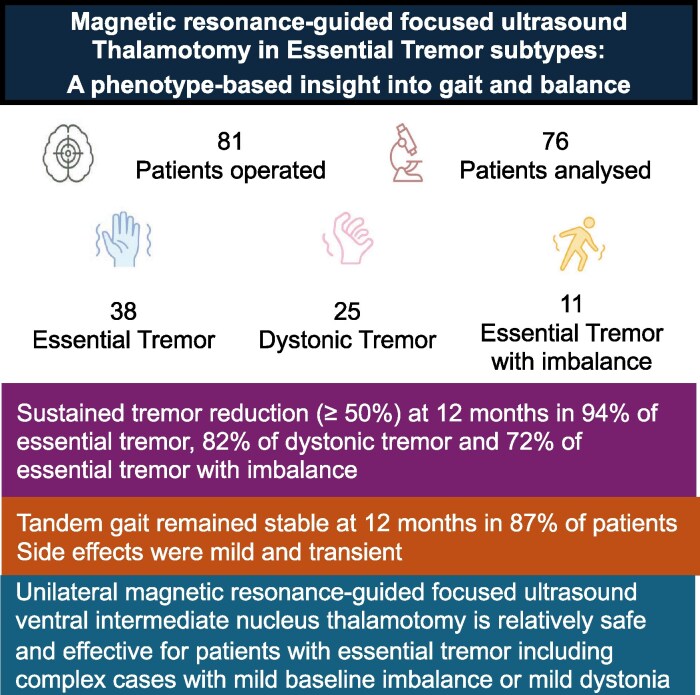

## Introduction

The clinical definition of essential tremor (ET) has evolved in recent years, particularly with the adoption of the new MDS classification, which differentiates between ET, essential tremor plus (ETP), and dystonic tremor (DT).^1^ Although controversial, these phenotypic subgroups might represent different underlying pathophysiological mechanisms or more complex disease forms, with increasing evidence pointing to a combined cerebellar and basal ganglia network dysfunction, particularly in DT syndromes.^2,3^

The term ETP has been a matter of debate, as it may represent a disease stage rather than a distinct subtype.^4,5^ Resting tremor can emerge in long-standing ET, and tandem gait abnormalities or cognitive impairment have been associated with aging.^5^ In addition, the interpretation of subtle or questionable dystonia often depends on the examiner’s judgement, so the same case may be classified as ETP by one observer and as DT by another.^6-8^ This diagnostic uncertainty has relevant implications when selecting candidates for surgery and when interpreting treatment outcomes.

Evidence on the impact of tremor phenotypes on magnetic resonance-guided focused ultrasound (MRgFUS) thalamotomy outcomes remains limited.^9^ MRgFUS thalamotomy is recognized as a safe and effective treatment for medication-refractory tremor,^10-13^ but gait ataxia has emerged as one of the most frequently reported treatment-related adverse events.^14,15^ However, no prior studies have thoroughly investigated whether baseline gait abnormalities or tremor phenotype modify the risk of gait worsening after treatment. This gap in knowledge is particularly relevant given that postural instability and tandem gait abnormalities are common in patients with ET and DT.^2,16^

Tremor severity and prevalence increase with age.^17,18^ Most individuals with ET are only mildly affected, but the condition can become highly disabling over time causing physical and psychosocial impairment despite optimal medical therapy.^18^ Unlike deep brain stimulation (DBS), MRgFUS does not apply strict upper age limits, which expands surgical eligibility and increases the proportion of older adults who may benefit from this procedure.^19^ Although MRgFUS thalamotomy represents a reasonable therapeutic option for selected elderly patients, age itself has also been consistently associated with poorer balance and gait performance.^5,16,20,21^ This fact reinforces the importance of understanding how these factors may affect surgical outcomes and long-term safety, particularly when considering treatment in this elderly population.

The primary outcome, defined *a priori*, was to assess whether gait and balance outcomes after MRgFUS thalamotomy differed according to tremor phenotype and baseline gait status. Additionally, we examine potential differences in treatment efficacy and adverse events across tremor subtypes. Our aim is to provide clinically meaningful evidence to support more individualized patient selection and informed decision-making, particularly for older patients or those with complex tremor phenotypes and pre-existing gait instability.

## Materials and methods

### Patient population

Eighty-one patients underwent unilateral MRgFUS thalamotomy targeting the ventral intermediate nucleus (VIM) of the thalamus between May 2019 and September 2022 at Hospital Universitario y Politécnico La Fe, Valencia, Spain. Patients with medically refractory tremor causing significant impairment in daily function were referred to our clinic, where they underwent prospective clinical evaluation and video recording. The indication for MRgFUS was determined independently of this study, following established criteria previously described: patients with postural or intention tremor of the hand scoring two or more on the Fahn–Tolosa–Marin clinical rating scale for tremor (CRST) and a skull density ratio (SDR) of 0.4 or higher.^10,22^

Patients were diagnosed according to the 2018 MDS Consensus criteria;^1^ however, for this study, a modified classification was used to better reflect the clinical aim of assessing gait and balance outcomes after MRgFUS VIM thalamotomy. Patients fulfilling criteria for ET but presenting impaired tandem gait at baseline—defined as more than one out-of-line misstep on a 10-step tandem gait^21,23^—were designated as ET with imbalance (ET-I), whereas those showing mild but clearly discernible dystonic posturing of the fingers and/or mild cervical dystonia were classified as DT. Because dystonia in these cases was mild, specific clinical signs were carefully assessed to ensure accurate identification. These included dystonic posturing of the fingers when the arms were outstretched, the presence of mirror movements, coexisting mild head tilt, and a multidirectional tremor axis when drawing four Archimedes spirals.^1,24,25^ Patients with questionable dystonic posturing—without additional dystonic features—or those with non-disabling rest tremor in the absence of parkinsonian signs were retained within the ET group. Exclusion criteria included clinically prominent dystonia features; parkinsonian signs at baseline, specifically bradykinesia or rigidity; evident cognitive impairment or dementia on clinical evaluation; and severe gait impairment requiring walking assistance. Mild balance abnormalities, including tandem gait disturbances, were not considered exclusion criteria for MRgFUS VIM thalamotomy.

Phenotype determination was based on the independent evaluation of preoperative video recordings by two movement disorder specialists and supplemented with medical records and paper charts for unrecorded aspects of the examination (e.g. spiral drawings, handwriting, rigidity). Substantial inter-rater agreement was achieved (κ & 0.72). In cases of diagnostic disagreement, a joint re-evaluation session was conducted to reach a final consensus diagnosis.

The study was conducted in accordance with the Declaration of Helsinki and was approved by the local Institutional Review Board. Written informed consent was obtained from all participants. The study was not prospectively registered, as it formed part of an ongoing institutional clinical programme.

### Focused ultrasound thalamotomy

The details of the MRgFUS VIM thalamotomy procedure have been previously published.^22^ All procedures were performed using the Exablate Neuro system (Insightec) under 3 T MRI guidance with real-time thermometry, as described in our previous report.^22^ Briefly, the VIM nucleus, contralateral to the most affected hand, was indirectly targeted on the preoperative MRI scan using traditional stereotactic planning. The target was defined as 14–15 mm from the midline or, alternatively, 11–11.5 mm from the wall of the third ventricle, 6–7 mm anterior to the posterior commissure and 2 mm above the intercommissural line. In all cases, patient-specific diffusion MRI tractography was used to delineate the dentato-rubro-thalamic (DRTT) and corticospinal tracts, allowing refinement of the mediolateral (*y*-axis) coordinate to optimize DRTT engagement while preserving a 4-mm safety margin from the pyramidal tract. The z-coordinate was kept 2 mm above the intercommissural line in all patients, except when an adjustment was required due to limited intraoperative tremor benefit. Treatment failure was defined as the inability to achieve at least one ablative sonication reaching an average temperature above 54°C and the absence of a visible lesion on the immediate post-treatment MRI. Patients who did not meet this criterion were excluded from further analysis. The total ablated VIM volume was measured by manual segmentation from axial, coronal and sagittal T_2_-weighted MRI slices on the immediate post-treatment 3D scan.

### Outcome assessments

Tremor type and severity were assessed using the CRST, focusing on the hand tremor score (HTS) for the treated side (subsections A and B; range: 0–32). Functional disability was evaluated with subsection C of the scale (range: 0–28). At each visit, patients were asked to estimate their percentage of self-reported improvement compared to baseline.

A comprehensive evaluation of gait and balance was also performed. The Berg balance scale (BBS) was used as a general assessment tool, given its widespread validation for balance disorders.^26^ However, based on our previous findings where the BBS remained stable post-treatment despite subtle postural changes,^22^ we included a more detailed tandem gait analysis to detect lateralized imbalance potentially related to the treated side. Patients were asked to walk 10 consecutive tandem steps, and the number of missteps was recorded.^21,23^ Tandem gait was classified as mildly impaired when patients made two or more missteps, moderately impaired when tandem gait could not be performed unassisted and severely impaired when assistance was required for walking. For patients diagnosed with DT, a preoperative assessment using the Burke–Fahn–Marsden dystonia rating scale (BFMDRS) was performed. All evaluations including CRST, BBS, tandem gait and BFMDRS were video recorded. These recordings were also used to establish the phenotypic classification. Two movement disorder specialists independently reviewed all video material. Clinical assessments were conducted at baseline and repeated at 6 and 12 months post-treatment. To provide a clinically meaningful measure of durability, we quantified the proportion of patients achieving a 50% improvement or higher, as well as those reaching a 75% improvement or higher, in HTS at 12 months. All evaluations were performed by neurologists specialized in movement disorders. Some patients were unable to attend in-person visits due to the COVID-19 pandemic, long travel distances or loss of contact despite multiple phone and written attempts. In such cases, a structured telephone interview was conducted, when possible, to assess adverse events and subjective tremor improvement.

Safety assessments included the systematic recording of adverse events during the procedure and at all follow-up visits, including the 1-month evaluation, to document both early postoperative changes and longer-term evolution. Adverse events were classified according to criteria previously established by our group, based on the Common Terminology Criteria for Adverse Events:^22,27^ mild (minor inconvenience, not affecting daily routine activities), moderate (bothersome, interfering with routine activities) or severe (incapacitating, limiting activities of daily living). Each adverse event was further categorized as sonication-related or thalamotomy-related.^22^

### Statistical analysis

Descriptive statistics were used to summarize baseline characteristics and clinical outcomes. Continuous variables were expressed as mean (±SD) or median (IQR), depending on normality (Kolmogorov–Smirnov test). Categorical data were presented as absolute and relative frequencies. Between-group comparisons were performed using one-way ANOVA or Kruskal–Wallis for continuous variables and chi-square or Fisher’s exact test for categorical variables. Longitudinal changes were analysed with linear mixed-effects models (random intercepts), including time as a repeated factor and adjusting for age and sex. For normally distributed outcomes, general linear models assessed main effects and time × phenotype interactions with Bonferroni correction for multiple comparisons. For non-normal distributions, Brunner–Langer models and ANOVA-type statistics were applied. Wilcoxon and Mann–Whitney U tests were used for pairwise comparisons and McNemar’s test for changes in adverse events over time. Model assumptions were verified using residual plots and formal tests. All tests were two-tailed, with α = 0.05. Exact *P*-values and 95% CIs are reported where relevant. To evaluate predictors of gait deterioration, a multivariable logistic regression model was performed to examine whether baseline gait status, age, sex, disease duration or tremor phenotype were associated with tandem gait worsening at follow-up.

No formal sample size calculation was performed a priori due to the exploratory nature of the study and the absence of prior data addressing phenotype-related gait outcomes after MRgFUS. This approach is consistent with similar phenotype-based MRgFUS cohorts,^9^ supporting the adequacy of the present sample. A post hoc power analysis indicated >99% power to detect within-subject effects and >90% for between-group effects (f = 0.33; α = 0.05), confirming sufficient statistical sensitivity. Missing data were handled by complete case analysis, without imputation.

Analyses were performed using SPSS version 15.0 (IBM Corp., Armonk, NY, USA) and R version 4.3.1 (R Core Team, 2023). Given the observational design, no blinding or randomization was applied.

## Results

### Patient characteristics and procedural parameters

**Figure 1:**
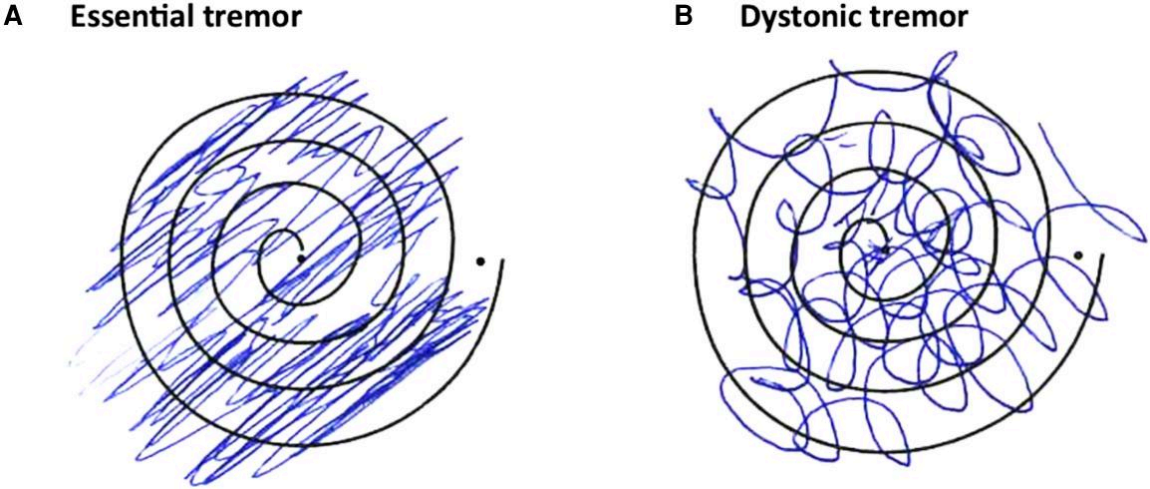
**Spiral drawings illustrating tremor axis patterns in essential tremor and DT.** Spirals drawn with the treated hand before treatment in one patient with essential tremor (**A**) and one with DT (**B**). In essential tremor, the tremor axis follows a consistent unidirectional pattern (8–2 o’clock). In DT, the spiral shows multidirectional deviations, suggesting co-contraction of proximal and distal muscles and variable tremor direction.

Of the 81 patients included in the study, 40 (49.4%) met criteria for ET; 11 (13.6%) were classified as ET-I, because they presented with ET-like tremor and impaired tandem gait; and 30 (37.0%) were diagnosed with DT as they exhibited mild dystonic posturing of the fingers when the arms were outstretched, with BFMDRS scores of five or less. Head tremor was present in 17 DT patients (56.7%), whereas only 7 (23%) showed mild cervical dystonia, primarily laterocaput or torticaput. Tremor was the predominant feature in all DT patients, and all of them except one displayed a multidirectional tremor axis in spiral drawings, contrasting with the unidirectional pattern observed in ET and ET-I patients. Figure 1 illustrates representative spiral patterns for ET and DT. The median age of the cohort was 73 years (range: 45–86 years), and the median disease duration was 20 years (range: 3–61 years). Patients with ET-I were the oldest (median 77 years, range 69–86), had the shortest disease duration (median 11 years, range 8–25), and exhibited the most severe disease burden (CRST score mean ± SD: 67.2 ± 11.9). Overall, 48 patients were male (59.3%), whereas DT patients were predominantly female (*n* = 17, 56.7%).

Regarding gait and balance, ET patients did not present tandem gait disturbances by definition. Among ET-I patients, mild tandem gait difficulty was present in six (54.5%), while the remaining five (45.5%) showed moderate impairment, being unable to perform tandem gait but not requiring assistance to walk. Nearly half of the DT patients (*n* & 14, 46.7%) also exhibited some degree of gait impairment prior to treatment, either mild (*n* & 7, 50%) or moderate (*n* & 7, 50%). In both DT and ET-I patients, gait impairment was predominantly characterized by difficulty performing tandem gait, reflecting subtle balance dysfunction rather than overt truncal or limb ataxia. Overall, considering all ET-I patients and the subset of DT patients with tandem gait abnormalities, 26 individuals (32.1%) presented with mild to moderate gait impairment at baseline.

All patients had a SDR of 0.45 or higher (0.58 ± 0.09), and the VIM was the surgical target in all cases (left side in 88.9%). Each patient received a mean of 6.8 sonications, and the mean peak target temperature during the final sonication was 58.3°C. In five patients (three DT and two ET), despite delivering more than 30 000 joules of energy, using power above 800 W, and applying sonication durations of at least 60 s, the target temperature of 55°C was not reached during any sonication, preventing effective ablation. These five patients were excluded from further analysis. The remaining 76 patients (38 ET, 11 ET-I and 27 DT) underwent at least one effective sonication. The average ablated VIM volume was 116.8 ± 36.1 mm^3^, as measured on immediate post-treatment MRI. Tremor improvement was achieved in all these cases. Demographic characteristics and treatment parameters are detailed in Table 1.

**Table 1:** Patient characteristics and treatment parameters across tremor phenotypes

Characteristics	Total (*n* = 81)	ET (*n* = 40)	ET-I (*n* = 11)	DT (*n* = 30)	Significance between groups (*P*-value)
Age at surgery (years)	73 (IQR:66–78)	68.5(IQR:60.5–75.5)	77(IQR:74–80)	74.5(IQR:67–80)	0.011**
Male gender	48 (59.3%)	29 (72.5%)	6 (54.5%)	13 (43.3%)	0.046*
Disease duration (years)	20 (IQR:10–34)	22.5 (IQR:11–38.5)	11 (IQR:10–20)	19.5 (IQR:10–37)	0.097
Treated HTS at baseline	18.7 ± 5.6	17.3 ± 5.0	22.9 ± 3.9	19.0 ± 6.1	NS
Total CRST at baseline	55.1 ± 16.4	49.5 ± 15.2	67.2 ± 11.9	58.1 ± 16.7	NS
Disability at baseline	16 (IQR:12.5–18)	14 (IQR:10–17)	19 (IQR:17–22)	17 (IQR:14–18)	NS
Impaired tandem gait (*n*)^a^	26 (32.1%)	0 (0%)	11 (100%)	14 (46.7%)	<0.001***
BBS score	54.5 ± 2.0	55.5 ± 0.09	51.6± 1.5	54.1 ± 2.2	0.004**
VIM thalamotomy (left)	72 (88.9%)	35 (87.5%)	11 (100%)	26 (86.7%)	NS
SDR	0.58 ± 0.09	0.59 ± 0.09	0.57 ± 0.08	0.56 ± 0.08	NS
Thalamic ablation^b^	76 (93.8%)	38 (95%)	11 (100%)	27 (90%)	NS
Average temperature^b^	58.3 ± 2.1(*n* = 76)	58.4 ± 1.9 (*n* = 38)	58.9 ± 1.5 (*n* = 11)	57.9 ± 2.4 (*n* = 27)	NS
Number of sonications^b^	6.8 ± 2.4 (*n* = 76)	6.9 ± 2.7 (*n* = 38)	6.7 ± 1.6 (*n* = 11)	6.7 ± 2.3 (*n* = 27)	NS
Lesion volume^b^	116.8 ± 36.1 (*n* = 76)	121.5 ± 44 (*n* = 38)	105.2 ± 17.9 (*n* = 11)	117.8 ± 33.9 (*n* = 27)	NS

Abbreviations: BBS, Berg balance scale; CRST, clinical rating scale for tremor; DT, dystonic tremor; ET, essential tremor; ET-I, essential tremor with imbalance; HTS, hand tremor score; NS, non-significant; SDR, skull density ratio.

Data are presented as mean ± standard deviation (SD), median (interquartile range, IQR) or number (percentage), as appropriate.

^a^Tandem gait performance was classified as follows: no impairment (0–1 misstep), mild (≥2 misstep), moderate (unable to perform) and severe (requiring walking aid).

^b^Five patients, who did not reach a maximum temperature of 55°C in at least one sonication and showed no visible lesion on the immediate post-treatment MRI, were excluded from subsequent analyses.

*P*-values refer to comparisons across the three phenotype groups using ANOVA (normal continuous variables), Kruskal–Wallis (non-normal continuous variables) or chi-square/Fisher’s exact tests (categorical): *P < 0.05; **P < 0.01; ***P < 0.001.

At baseline, significant differences between groups were observed in age (*P* = 0.011), sex distribution (*P* = 0.046), BBS scores (*P* = 0.004) and tandem gait performance (*P* < 0.001). A trend towards shorter disease duration was also noted in patients with ET-I (*P* = 0.097). However, no significant differences were found among phenotypes in terms of tremor severity, treatment parameters or ablation volume (see Table 1). Likewise, although not analysed in detail, the five patients in whom effective thermal ablation was not achieved did not differ meaningfully from the rest of the cohort.

A flow chart detailing patient inclusion, exclusions and follow-up completion for each phenotype is provided in Supplementary Fig. 1.

### Tremor and functional improvement

Of the 76 patients in whom thermal ablation was successfully achieved, 73 (37 ET, 25 DT, 11 ET-I) attended the 6-month follow-up visit, and 61 (32 ET, 22 DT, 7 ET-I) were evaluated at 12 months and thus included in the tremor outcome analysis. No significant differences in baseline characteristics or surgical parameters were observed between patients who completed follow-up and those who did not.

In the overall cohort, significant improvements were observed in the HTS, total CRST, and disability scores at both 6 and 12 months (*P* < 0.001 for all comparisons). Self-reported improvement was marked (≥75%) in 96% of patients at 6 months and remained high at 12 months (85%). Subgroup analysis revealed that all phenotypes showed significant clinical improvement (*P* < 0.01), with no differences in the magnitude of HTS reduction between groups at either follow-up. HTS improved by 80% in ET, 70% in ET-I and 71% in DT at 6 months and remained significantly improved at 12 months (ET, 78%; ET-I, 63%; DT, 63%). However, at the final visit, a modest increase in mean HTS was observed in ET-I (δ, +2.14; 95% CI: 0.37 to 4.66; *P* = 0.045) and DT (δ, +1.0; 95% CI: 0.49 to 2.29; *P* = 0.038) compared to their respective 6-month scores, while ET remained stable throughout follow-up. Sustained response (≥50%) was observed in 94% of ET patients, 82% of DT patients and 71% of ET-I patients. A more stringent threshold (≥75%) was met by 66% of ET patients, 27% of DT patients and 43% of ET-I patients. An individual-trajectory plot illustrating HTS evolution for each phenotype is shown in Supplementary Fig. 2. A similar pattern was observed for total CRST and disability scores. In ET patients, total CRST improved by 57% at 6 months and remained stable at 12 months (53%; *P* < 0.001). ET-I and DT patients showed marked improvement at 6 months (ET-I: 49%, *P* < 0.001; DT: 53%, *P* < 0.001), followed by significant worsening at 12 months (ET-I: δ + 10.4, 95% CI 1.71–19, *P* & 0.014; DT: δ + 5.0, 95% CI 0.3–9.7, *P* & 0.034), although scores remained better than baseline. Disability scores showed the same evolution: large initial gains in all groups, stable in ET, but with partial loss of benefit at 12 months in ET-I and DT. Table 2 provides detailed baseline values and longitudinal changes in HTS, CRST and disability scores for each phenotype, including adjusted mean differences with their 95% CIs.

**Table 2:** Baseline values and longitudinal changes in tremor severity and disability across phenotypes, including adjusted mean differences with 95% confidence intervals

Phenotype		Baseline	6 months	Mean difference (95% CIs)	Significance within-group (*P-*value) (6 months)	12 months	Mean difference (95% CIs)	Significance within-group (*P-*value) (12months)
Total Cohort	No.	76	73			61		
	Treated HTS	18.7 ± 5.6	4.7 ± 3.6		<0.001***	5.4 ± 5.0		<0.001***
	CRST	55.1 ± 16.4	25.2 ± 11.5		<0.001***	28.8 ± 15.4		<0.001***
	Disability	16 (IQR:12.5–18)	2 (IQR:0–3)	NA	<0.001***	1 (IQR: 1–4)	NA	<0.001***
ET	No.	38	37			32		
	Treated HTS	17.3 ± 5.0	3.4 ± 2.6	14.2 (12–16.4)	<0.001***	4.0 ± 4.6	14.4(11.8–16.9)	<0.001***
	CRST	49.5 ± 15.2	21.3 ± 10.7	28.5 (23.5–33.4)	<0.001***	23.4 ± 14.3	27.6 (21.4–33.8)	<0.001***
	Disability	14 (IQR:10–17)	1 (IQR:0–2)	NA	<0.001***	1 (IQR:0–2)	NA	<0.001***
ET-I	No.	11	11			7		
	Treated HTS	22.9 ± 3.9	6.8 ± 4.4	16.4 (11.8–21)	<0.001***	7.7± 6.7	14.3 (9.1–19.5)	<0.001***
	CRST	67.2 ± 11.9	34.1 ± 10.5	27 (14.6–39.4)	<0.001***	39.1 ± 15.6	16.6 (1.2–32)	=0.031*
	Disability	19 (IQR:17–22)	3 (IQR:1–9)	NA	=0.009**	3 (IQR:2–14)	NA	=0.054
DT	No.	27	25			22		
	Treated HTS	19.0 ± 6.1	5.7 ± 3.8	14.2 (11.5–17)	<0.001***	6.9 ± 4.5	13.2 (10.2–16.3)	<0.001***
	CRST	58.1 ± 16.7	27.2 ± 10.7	32 (25.3–38.7)	<0.001***	33.3 ± 14.4	27 (18.6–35.4)	<0.001***
	Disability	17 (IQR:14–18)	3 (IQR:1–6)	NA	<0.001***	2.5(IQR:1–8)	NA	<0.001***

Abbreviations: CRST, clinical rating scale for tremor; DT, dystonic tremor; ET, essential tremor; ET-I, essential tremor with imbalance; HTS, hand tremor score; NA, not applicable; No., number of patients.

Data are presented as mean ± SD or median (IQR), as appropriate. Mean differences (95% CIs) represent adjusted changes from baseline to 6 and 12 months.

*P*-values correspond to within-group longitudinal comparisons using linear mixed-effects models or Brunner–Langer models for non-normal data, *P < 0.05; **P < 0.01; ***P < 0.001.

Between-group differences were assessed with ANOVA (normal continuous variables) or Kruskal–Wallis tests (non-normal continuous variables), with no significant phenotype-related differences at any time point.

**Figure 2:**
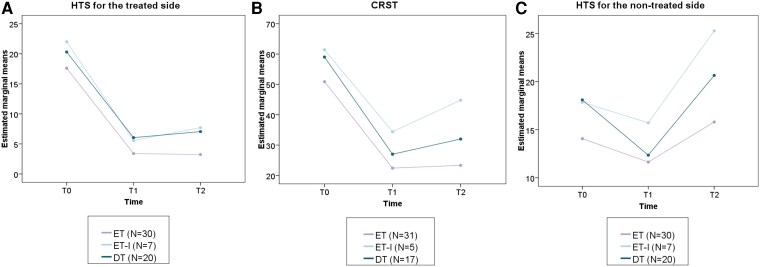
**Evolution of tremor scores across phenotypes at baseline, 6 months and 12 months.** Adjusted mean tremor scores are shown for ET, ET-I and DT at baseline (T0), 6 months (T1) and 12 months (T2). Values represent model-estimated marginal means derived from linear mixed-effects models adjusted for age and sex. Panel A displays the HTS for the treated side; panel B the total CRST score and panel C the HTS for the non-treated hand. Each data point represents the adjusted mean value for the corresponding group and time point. HTS and CRST improved significantly from baseline in all phenotypes (*P* < 0.01). No significant differences between phenotypes were observed at any follow-up. A mild increase in tremor scores at 12 months was observed in ET-I and DT compared with 6 months (*P* = 0.045 and *P* = 0.038, respectively), while ET remained stable. Non-treated hand tremor increased over time in ET-I patients (*P* < 0.005).

Age- and sex-adjusted models confirmed a positive correlation between increasing age and greater tremor severity (HTS, *P* = 0.001; CRST, *P* = 0.007). However, no significant time × phenotype × age interaction was detected, confirming that age did not differentially influence longitudinal treatment response across phenotypes. Interestingly, in the untreated hand, ET and DT patients showed significant improvement at 6 months (*P* < 0.001), followed by a return to baseline at 12 months. In contrast, ET-I patients exhibited no benefit at 6 months and a significant seven-point worsening at 12 months compared with baseline (*P* < 0.005). Age-adjusted models showed that untreated hand tremor was strongly associated with age (F = 12.7; *P* = 0.001), and this relationship varied over time, as indicated by a significant time × age interaction (F = 3.27, *P* = 0.046), with older patients demonstrating greater deterioration. See Fig. 2 for further detail.

### Adverse events

Intraoperative adverse events were reported in 50% of patients, with dizziness—commonly described as a sensation of ‘lightness’—and nausea being the most frequently observed. All intraoperative adverse events resolved immediately after the procedure and did not lead to treatment interruption.

Regarding post-thalamotomy adverse events—excluding gait disturbances, which are analysed separately—60% of patients who completed both follow-up visits did not report any event. Among the 40% who experienced adverse events during follow-up, all were rated as mild. The most frequently reported event at both 6- and 12-month evaluations was paraesthesia in the mouth or in the fingers of the treated hand (28% and 16%, respectively). Mild lower-limb weakness was present at 6 months in 3 patients (4%); at 12 months, it persisted in one patient, improved in another, and follow-up data were missing for the third. Other adverse events at 12 months were infrequent and non-disabling, including dysmetria in five patients (8%) and dysgeusia and dysarthria in two patients each (3%). Notably, both paraesthesia and dysarthria significantly improved over time (*P* = 0.045 and *P* = 0.019, respectively), suggesting that these effects tend to resolve in most patients. In contrast, weakness and dysmetria, although uncommon, appeared more likely to persist over time.

No significant differences in adverse events frequency or severity were observed across tremor phenotypes. Importantly, no DT patient experienced a clear worsening of dystonia after the procedure, although subtle changes cannot be definitively excluded.

A detailed overview of adverse events at both early (1-month) and longer-term (6- and 12-month) follow-up, reported for the full cohort and stratified by phenotype, is available in the Supplementary Table 1.

### Gait and balance

The three patients who presented with lower-limb weakness at 6 months were excluded from the gait analysis. Table 3 summarizes gait and balance outcomes according to the tremor phenotype.

**Table 3:** Longitudinal evolution of balance and tandem gait performance across tremor phenotypes

Phenotype		Baseline	6 months	12 months	Significance within-group (*P*-value)
Total Cohort	No.	76	70	60	NS
	BBS score	54.5 ± 2	54.3 ± 2.3	53.7 ± 3.5	NS
	tandem gait worsening from baseline^a^	NA	11 (15.7%)	8 (13.3%)	NS
ET	No.	38	35	31	NS
	BBS score	55.5 ± 0.9	55.5 ± 1	54.8 ± 3.3	NS
	tandem gait worsening from baseline^a^	NA	5 (14.3%)^b^	3 (10%)	NS
ET-I	No.	11	11	7	NS
	BBS score	51.6 ± 1.5	51.9 ± 2.3	50.0 ± 3.6	NS
	tandem gait worsening from baseline^a^	NA	1 (9.1%)	1 (14.3%)	NS
DT	No.	27	25	22	NS
	BBS score	54.1 ± 2.2	53.4 ± 2.6	53.3 ± 3.1	NS
	tandem gait worsening from baseline^a^	NA	5 (20%)	4 (18.1%)	NS

Abbreviations: BBS, Berg balance scale; DT, dystonic tremor; ET, essential tremor; ET-I, essential tremor with imbalance; NA, not applicable; No., number of patients; NS, non-significant.

BBS scores are shown as mean ± standard deviation (SD).

^a^Tandem gait performance is reported as the number (percentage) of patients who worsened at the different time points compared to baseline, with worsening defined as a change from no impairment to mild, moderate or severe impairment, or as progression within categories. NA indicates that worsening cannot be defined at baseline. Three patients were excluded from tandem gait analysis due to lower-limb weakness that could interfere with test interpretation.

*P*-values for within-group comparisons versus baseline were obtained using linear mixed-effects models or Brunner–Langer models. Between-group differences were assessed with ANOVA (normal continuous variables) or Kruskal–Wallis tests (non-normal continuous variables) and chi-square/Fisher tests (categorical), with no significant differences between phenotypes at any follow-up.

^b^Non-significant trend in ET at 6 months; *P* = 0.075.

At baseline, ET patients had significantly higher BBS scores than those with ET-I or DT, as expected. These differences persisted across follow-up, without significant decline in any group. As previously reported, BBS scores were negatively associated with age (*P* = 0.006), but age-adjusted models confirmed that this effect was consistent over time and across phenotypes.

**Figure 3:**
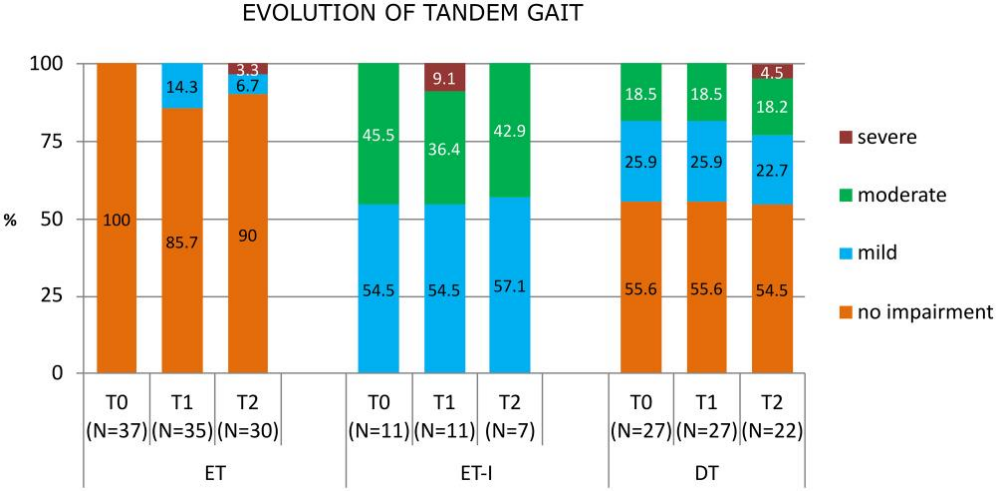
**Evolution of tandem gait performance across tremor phenotypes.** Stacked bars represent the distribution of tandem gait impairment severity in ET, ET-I and DT at baseline (T0), 6 months (T1) and 12 months (T2). Tandem gait was categorized as no impairment (0–1 missteps during 10 tandem steps), mild (two or more missteps), moderate (unable to perform the test) and severe (walking assistance required). The absolute number of patients contributing data at each time point is displayed below the corresponding bars.

Tandem gait was specifically analysed to detect subtle changes. Baseline differences remained stable throughout follow-up, and no group showed significant deterioration. At 6 months, a non-significant trend towards mild worsening was observed in ET patients (*P* = 0.075), with 14% (*n* = 5) presenting at least two missteps. Similar mild worsening occurred in ET-I (*n* = 1, 9%) and DT (*n* = 5, 20%), without reaching statistical significance. At 12 months, tandem gait remained stable in 87% of patients, and no significant differences emerged between groups. Age was correlated with tandem gait difficulty (*P* = 0.002), but longitudinal changes were not influenced by age or tremor subtype. Although not statistically significant, it is noteworthy that all patients who experienced tandem gait worsening at 12 months (ET, *n* = 3; ET-I, *n* = 1; DT, *n* = 4) were over 73 years old. At the last follow-up, only two elderly patients showed severe gait impairment. The first was a woman with ET whose tremor began at age 59. At baseline (age 74), she exhibited severe postural and action tremor with intermittent mild rest tremor in both hands, without rigidity or bradykinesia. Tandem gait was normal (BBS: 56). At 6 months, tandem gait remained intact (BBS: 56), but by 12 months she developed marked worsening of left-hand rest tremor accompanied by ipsilateral rigidity and bradykinesia. Additionally, her BBS score declined to 52, and she required a cane for ambulation. A DAT-SPECT performed at that time confirmed a diagnosis of Parkinson’s disease, and initiation of levodopa therapy improved gait and balance. The second case was an 82-year-old woman with DT beginning in adolescence, with associated head tremor and dystonic voice tremor. At baseline, she was already unable to perform tandem gait (BBS: 51). Her stability remained largely unchanged at 6 months (BBS: 50), but by 12 months she required a walking aid and showed a marked decline in balance (BBS: 42). Moreover, one patient with ET-I who exhibited severe tandem gait worsening at the 6-month visit did not attend the 12-month follow-up due to an unrelated medical condition. This patient was also over 80 years old. Notably, four patients (1 ET-I, 3 DT) improved over time, shifting from moderate to mild instability. Figure 3 displays the evolution of tandem gait performance across tremor phenotypes.

In addition to objective assessments, patients were systematically asked about subjective unsteadiness, which was significantly associated with older age (*P* & 0.026), regardless of objective performance or tremor phenotype.

Multivariable logistic regression analysis confirmed that no baseline variable—including age, phenotype, sex, disease duration or baseline gait status—was significantly associated with an increased risk of tandem gait worsening at follow-up. Overall, gait and balance deterioration was mild, observed in 11 patients (15.7%) at 6 months and 8 patients (13.3%) at 12 months.

## Discussion

The present study addresses an important and previously underexplored question: whether gait and balance outcomes after unilateral MRgFUS VIM thalamotomy differ depending on tremor phenotype or baseline gait and balance status. Given the growing concern regarding post-thalamotomy ataxia, particularly in patients with baseline instability, this represents a clinically meaningful endpoint with direct implications for patient selection and risk stratification. To facilitate clinical interpretation, a pragmatic phenotype classification was used, grouping patients with tandem gait impairment as ET-I, those with mild but clear dystonic features as DT, and retaining non-disabling rest tremor cases without parkinsonian signs within the ET group.

Our findings demonstrate that, although patients with ET-I and DT exhibited significantly worse baseline BBS scores and tandem gait performance than those with ET without imbalance, unilateral MRgFUS VIM thalamotomy did not result in meaningful gait or balance deterioration across phenotypes. Tandem gait remained stable in most patients at 12 months, regardless of tremor phenotype or preoperative gait status, and only 10–15% developed mild to moderate worsening. At the last follow-up, only two elderly patients developed severe gait impairment. One was subsequently diagnosed with Parkinson’s disease despite having no parkinsonian signs at baseline. This case most likely reflects disease evolution rather than a direct surgical effect, as a subset of patients may present with long-standing ET that later evolves into Parkinson’s disease.^28^ The second was a DT patient who already exhibited moderate instability at baseline. In this patient, the timing and gradual progression of balance impairment likewise suggest deterioration of the underlying disorder rather than an adverse effect of the thalamotomy. Notably, no clinical worsening of dystonia was observed, and the pattern of gait decline was more consistent with increasing instability related to cerebello-thalamo-cortical dysfunction than with a dystonic mechanism.^2,3^ Interestingly, some patients with ET-I and DT showed post-treatment improvement, shifting from moderate to mild tandem gait instability. This may reflect indirect benefits from reduced axial or lower-limb tremor, as these patients had high baseline CRST scores. Overall, neither baseline balance impairment, age nor phenotype was associated with a higher risk of postoperative tandem gait worsening. These findings suggest that MRgFUS thalamotomy is generally well tolerated in terms of balance and gait, even in individuals with more complex tremor phenotypes, challenging the perception that these subgroups are at higher risk of functional deterioration. Still, caution is warranted in individuals with moderate or severe baseline deficits, as they were underrepresented in our sample.

Moreover, the inclusion of tandem gait analysis proved valuable, as BBS scores alone may not detect subtle lateralized imbalance. This methodological addition was informed by our prior findings^22^ and aligns with existing literature associating ET with poorer tandem gait performance.^16,20,21^ Importantly, although age was not associated with objective gait worsening, older patients more frequently reported persistent subjective imbalance. This perceived instability may reflect subclinical deficits not captured by the BBS or tandem gait assessments, particularly in older adults, where age-related decline in sensory integration, vestibular function or motor planning may contribute to balance impairment.^29^ Notably, although not statistically significant, all patients who experienced tandem gait worsening at 1 year were over 73 years old. Moreover, those who developed severe gait disturbances at either 6 or 12 months were also older than 73 regardless of tremor phenotype. Cognitive impairment has been shown to correlate with tandem gait disturbances,^30^ and both are known to increase with age.^5^ Although we did not perform formal cognitive testing, patients with evident cognitive impairment were excluded at baseline, and no consistent postoperative cognitive decline has been reported after VIM-MRgFUS.^31^ Although a contribution of cognitive impairment to gait performance in older patients in our cohort cannot be fully excluded, our findings highlight the need for a more comprehensive preoperative balance assessment in elderly candidates.

Our surgical approach placed the lesion centre 2 mm above the intercommissural line, following the findings of Boutet *et al*.,^32^ who demonstrated that more ventral or inferolateral thalamic lesions are associated with a higher risk of ataxia due to extension into cerebellar outflow pathways. This slightly more rostral placement likely reduced the probability of DRTT involvement, although formal analysis of lesion coordinates and volume was beyond the scope of the present study. This consideration may be particularly relevant in ET-I and DT patients, in whom greater cerebello-thalamo-cortical dysfunction has been described, suggesting increased vulnerability to balance alterations.^2,3,33^

Beyond gait and balance outcomes, unilateral MRgFUS VIM thalamotomy also proved effective in improving contralateral hand tremor regardless of ET subtype, consistent with prior VIM-DBS studies showing similar efficacy in both ET and non-pure ET patients.^34-36^ At 1 year, patients with ET without imbalance showed a 78% improvement in HTS and an 86% reduction in disability scores. These results are somewhat higher than those reported in previous MRgFUS series—50–60% improvement at 1 year.^10-13,37^ Differences in phenotype stratification, inclusion criteria, baseline severity, assessment timing or lesion characteristics may reasonably account for these discrepancies. In our study, resting tremor was not considered an exclusion criterion for ET, as it has been described in long-standing disease,^4,5^ and it was not the predominant disabling feature in any included patient. Moreover, all patients underwent comprehensive neurological examinations at baseline and follow-up to exclude Parkinsonian signs.

In the ET-I subgroup, clinical improvement at 6 months was comparable to the ET (HTS 70%, disability 76%), but a partial decline was observed at 12 months (63% and 61%, respectively), while benefits remained clearly superior to baseline. These findings align with Steffen *et al*.,^35^ who reported similar VIM-DBS outcomes in ET and ETP, although ETP patients required higher stimulation intensities to achieve comparable tremor suppression. Additionally, ET-I patients also showed a significant seven-point worsening in untreated hand tremor at 12 months, suggesting disease progression. Treatment response was not age-dependent across groups; however, worsening of tremor in the untreated hand among ET-I patients was clearly age-related (*F* = 12.7, *P* = 0.001), with older individuals showing more pronounced deterioration at follow-up. As previously described,^16,20,21^ age is associated with greater tremor severity and impaired tandem gait. Progressive cerebello-thalamo-cortical dysfunction, which worsens with age and correlates with reduced functional connectivity and tremor severity,^33^ may plausibly underlie the observed decline in older ET-I patients.

Patients with DT also experienced significant improvement in contralateral hand tremor following VIM-MRgFUS, with no differences compared to ET or ET-I at any follow-up point. At 6 months, average improvements were 71% in HTS and 77% in disability scores, although a reduction in effect was also noted at 12 months (63% and 72%, respectively). This decline, particularly in functional outcomes, has also been reported by Peters *et al*.,^9^ suggesting that long-term benefit in DT may be less sustained. In DT, tremor arises from co-contraction of agonist and antagonist muscles, often affecting both distal and proximal segments of the limb.^6^ While VIM targeting effectively suppresses distal tremor, its effect on proximal involvement may be more limited. Most of our DT patients exhibited multidirectional tremor in spiral drawings—suggestive of proximal muscle recruitment—as opposed to the unidirectional pattern seen in ET and ET-I,^25^ which may partly explain the decline in functional outcomes over time. The optimal surgical target for DT remains under discussion.^38,39^ Although most centres favour VIM over GPi when tremor is the primary disabling symptom,^9,36,40^ some studies have reported worsening or unmasking of dystonia after thalamic DBS or lesioning.^41-43^ Combined VIM and GPi stimulation has been proposed to address both tremor and dystonia components.^44^ In our series, no clinical worsening of dystonia was observed at 12 months. This likely relates to the mild dystonic phenotype of our cohort, as all DT patients had BFMDRS scores of five or less and tremor was the predominant disabling symptom. Nevertheless, treatment response and safety profiles may differ in patients with more pronounced dystonic components so our findings should not be extrapolated to those with more severe dystonia. Furthermore, the slight decline in disability scores may reflect persistence or progression of dystonic features. Importantly, recent evidence suggests that DT involves dysfunction of both the cerebello-thalamo-cortical and the basal ganglia-thalamocortical circuits.^2,3^ Tsuboi *et al*.^45^ showed that stimulation of pallidothalamic fibres projecting to the ventralis oralis posterior (VOp) was associated with tremor reduction in DT and proposed the VIM/VOp border as a promising target. Supporting this, recent MRgFUS studies in focal hand dystonia targeting the same region have also yielded positive outcomes.^46,47^ Together, these findings and our own suggest that VIM targeting remains a valid approach in DT when tremor is the most disabling symptom. However, in selected patients—particularly those with prominent dystonia or proximal tremor—a slight anterior shift of the lesion towards the VIM/VOp border may optimize outcomes. Further studies are needed to define the most effective and safest target in this complex phenotype.

Overall, at 12 months, sustained tremor reduction was maintained by most patients across all phenotypes, with 50% improvement or higher in 94% of ET patients, 82% of DT patients and 72% of ET-I patients. Taken together, the partial decline observed at 12 months in ET-I and DT likely reflects a combination of age-related disease progression and phenotype-specific network dysfunction. In addition, factors such as lower SDR, skull hyperostosis or age-related white-matter changes may theoretically reduce thermal efficiency,^48,49^ although these aspects were not specifically evaluated in this study. While lesion volume, SDR and targeting parameters did not differ significantly across phenotypes, the ET-I group was small, and the study was not powered to detect subtle volume–outcome associations. Consequently, we cannot exclude the possibility that individual differences in tract anatomy or lesion efficiency contributed to the variability in long-term outcomes. Future lesion–outcome mapping studies will be essential to further elucidate these relationships.

Interestingly, both ET and DT patients in our cohort showed a transient reduction in untreated hand tremor at 6 months. Although this effect was not sustained at 12 months, it raises the possibility of bilateral network modulation despite the unilateral nature of the lesion. To our knowledge, improvement of ipsilateral tremor following unilateral VIM thalamotomy has not been explicitly reported. Jameel *et al*. described bilateral tremor improvement after a dual-target MRgFUS approach involving both the VIM and the posterior subthalamic area;^50^ however, their results are not directly comparable to ours, as our procedures involved a single-target lesion. The anatomical organization of the DRTT—containing both decussating and non-decussating fibres—offers a plausible substrate for such bilateral network modulation.^45^ In the absence of direct clinical evidence, this remains a hypothesis that warrants further investigation through tractography and functional imaging.

This study has several limitations. First, the ET-I group was relatively small, which may have reduced the statistical power to detect significant differences in some analyses. To address this, we applied Bonferroni correction for multiple comparisons, thereby increasing statistical stringency and reducing the risk of false positives, although this may also have limited the ability to detect subtle differences. Second, the phenotypic overlap between ET, ETP and DT, particularly in patients with subtle dystonic features or rest tremor, remains a recognized source of diagnostic variability and should be considered when interpreting our results. However, the inter-rater agreement for phenotype classification was substantial, and the pragmatic adaptation of the MDS classification used in this study allowed us to explore the clinical impact of imbalance or mild dystonia on MRgFUS thalamotomy outcomes, which was the main purpose of the present study. Cognitive function was not formally assessed, which represents an additional limitation, as cognitive impairment may influence balance outcomes in older adults. Future studies should include standardized cognitive testing to clarify this relationship.

## Conclusion

Unilateral MRgFUS VIM thalamotomy appears to be a relatively safe and effective treatment for patients with ET, ET-I and DT when tremor is the primary disabling symptom and baseline gait impairment is mild. Tremor control was substantial across phenotypes, although patients with ET-I and DT showed a modest decline over time, suggesting phenotype-specific durability considerations. Older age may contribute to subjective imbalance and predispose to more persistent gait disturbances. These findings highlight the importance of phenotype-adapted patient selection and long-term follow-up and may inform future refinements in targeting strategies for complex tremor syndromes.

## Supplementary Material

fcag076_Supplementary_Data

## Data Availability

The code used for data processing, modelling and visualization is provided in the Supplementary materials, in accordance with Brain Communications data-transparency standards. Additional data are available from the corresponding author upon reasonable request.
