# Supplementary material for: Magnetic resonance-guided focused ultrasound thalamotomy in essential tremor subtypes: a phenotype-based insight into gait and balance
Source: Brain Commun. 2026 Mar 11;8(2):fcag076. doi: 10.1093/braincomms/fcag076 (PMC13022827; doi:10.1093/braincomms/fcag076)
Supplement: fcag076_Supplementary_Data [file fcag076_supplementary_data.docx]

**Supplementary Table 1. Adverse events throughout follow-up stratified by tremor phenotype**

| **Phenotype** |  | **1 month** | **6 months** | **12 months** |
| --- | --- | --- | --- | --- |
| **Total Cohort** | **No.** | 76 | 75 | 64 |
|  | **Dysarthria** | 8 (10.52%) | 3 (4%) | 2 (3.3%) |
|  | **Paresthesia** | 27 (35.52%) | 21(28%) | 10 (16%) |
|  | **Dysmetria** | 18 (23.7%) | 8 (10%) | 5 (8%) |
|  | **Weakness** | 6 (7.9%) | 3 (4%) | 1 (3.1%) |
|  | **Disgeusia** | 5 (6.6%) | 2 (2.7%) | 2 (2.7%) |
|  | **Subjective unsteadiness** | 44 (57.9%) | 31 (41.3%) | 22 (34.4%) |
| **ET** | **No.** | 36 | 37 | 32 |
|  | **Dysarthria** | 5 (13.9%) | 2 (5.4%) | 2 (6.2%) |
|  | **Paresthesia** | 15 (41.7%) | 9 (24.3%) | 4(12.5%) |
|  | **Dysmetria** | 11 (30.5%) | 4 (10.8%) | 2 (6.2%) |
|  | **Weakness** | 3 (8.3%) | 2 (5.4%) | 1 (3.1%) |
|  | **Disgeusia** | 1 (2.8%) | 0 (0%) | 0 (0%) |
|  | **Subjective unsteadiness** | 19 (52.8%) | 9 (24.3%) | 7 (21.9%) |
| **ET-I** | **No.** | 13 | 11 | 7 |
|  | **Dysarthria** | 1 (7.7%) | 0 (0%) | 0 (0%) |
|  | **Paresthesia** | 4 (30.8%) | 4 (36.6%) | 0 (0%) |
|  | **Dysmetria** | 2 (15.4%) | 1 (9.1%) | 0 (0%) |
|  | **Weakness** | 1 (7.7%) | 1 (9.1%) | 0 0(%) |
|  | **Disgeusia** | 2 (15.4%) | 0 (0%) | 0 (0%) |
|  | **Subjective unsteadiness** | 9 (69.2%) | 7 (63.6%) | 4 (57.1%) |
| **DT** | **No.** | 27 | 27 | 22 |
|  | **Dysarthria** | 2 (7.40%) | 1 (3.7%) | 0 (0%) |
|  | **Paresthesia** | 8 (29.6%) | 8 (29.6%) | 6 (27.3%) |
|  | **Dysmetria** | 5 (18.5%) | 3 (11.1%) | 3 (13.6%) |
|  | **Weakness** | 2 (7.4%) | 0 (0%) | 0 (0%) |
|  | **Disgeusia** | 2 (7.4%) | 2 (7.4%) | 2 (9.1%) |
|  | **Subjective unsteadiness** | 16 (59.2%) | 15 (55.5%) | 11 (50%) |

Adverse events recorded at 1 month, 6 months, and 12 months after MRgFUS VIM-thalamotomy, stratified by tremor phenotype. Values represent number (percentage) of patients among those assessed at each timepoint. Subjective unsteadiness refers to patient-reported imbalance not accompanied by measurable deterioration on tandem gait testing or Berg Balance Scale.

Adverse events were graded as: mild = minor inconvenience not affecting daily activities; moderate = interfering with daily activities; severe = incapacitating. At 1 month, all adverse events were rated as mild except for one patient with moderate ipsilateral weakness and one with moderate ipsilateral dysmetria. At 6 and 12 months, all adverse events were mild. No patient experienced a severe adverse event at any point during follow-up.

Abbreviations: DT, dystonic tremor; ET, essential tremor; ET-I, essential tremor with imbalance; No., number of patients


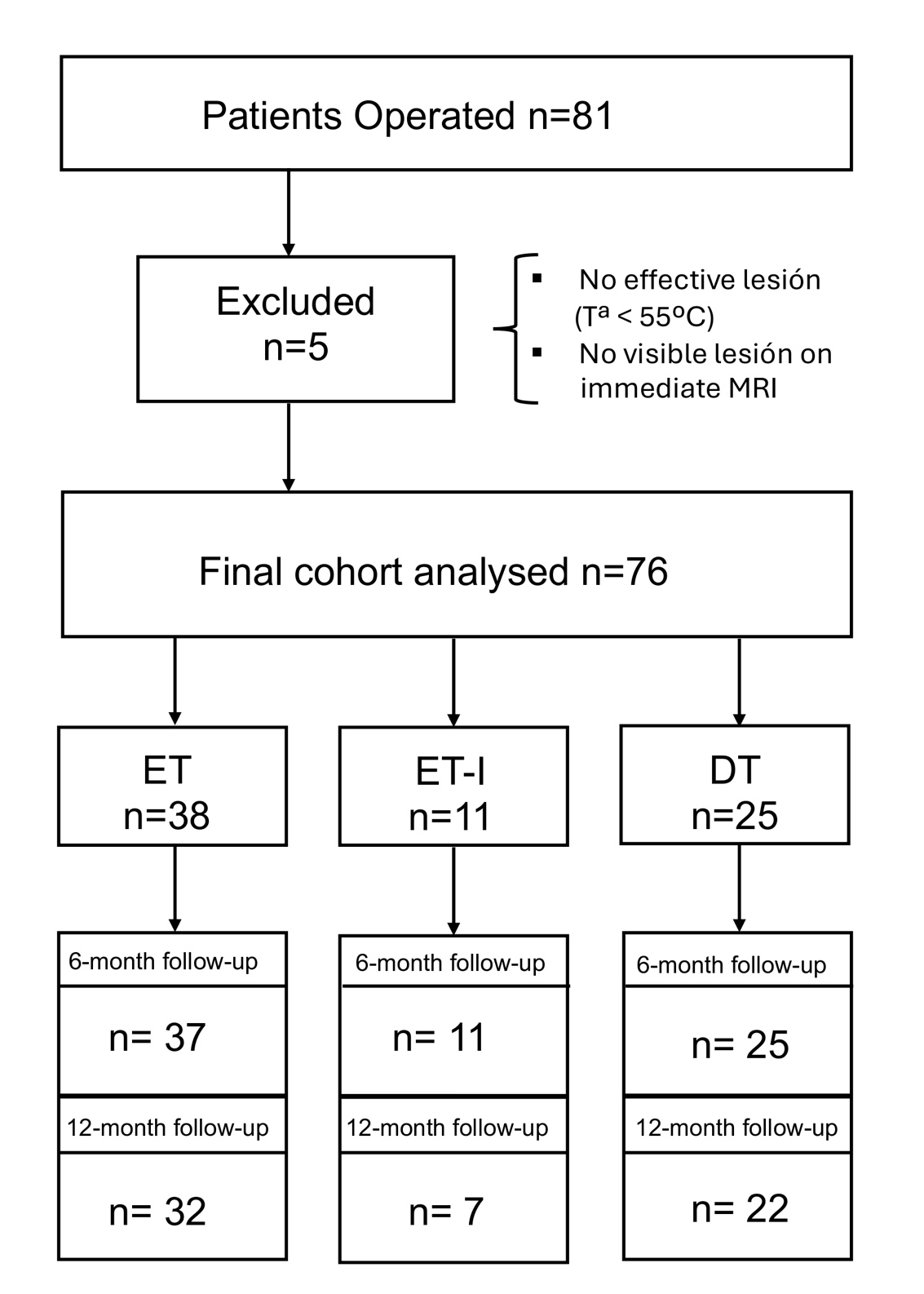


**Supplementary Fig. 1. Flow chart of patient inclusion, exclusions, and follow-up completion by phenotype.**

Flow diagram summarizing patient screening, exclusions, and follow-up completion. Of the 81 patients undergoing MRgFUS, five were excluded due to failure to achieve an effective lesion (no sonication ≥55 °C and/or absence of a visible lesion on immediate MRI). The final cohort comprised 76 patients: essential tremor (ET, n=38), essential tremor with imbalance (ET-I, n=11), and dystonic tremor (DT, n=25). Follow-up completion at 6 and 12 months for each phenotype group is shown.

**
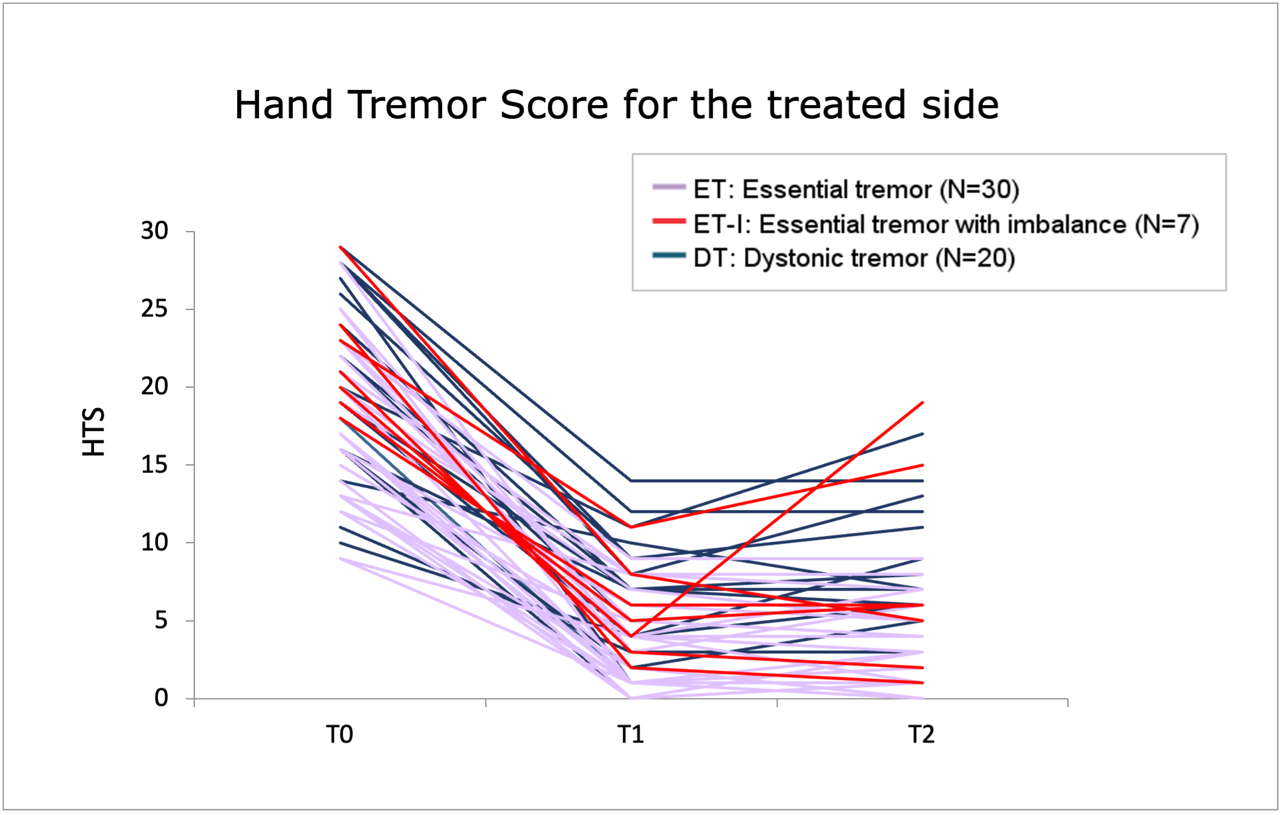
Supplementary Fig. 2.** **Individual trajectories of Hand Tremor Score (HTS) on the treated side.**

Each line represents one patient, illustrating individual HTS changes from baseline (T0) to 6 months (T1) and 12 months (T2). Colors correspond to tremor phenotypes: ET (light purple), ET-I (red), and DT (dark blue). The plot highlights both the magnitude of improvement at T1 and the subsequent stability or partial decline observed at T2 within each phenotype.

**Code for data Analysis**

*****************************************************.

***** code for analysis in SPSS**********.

*****************************************************.

GET

FILE='Data'.

CROSSTABS

/TABLES=SEXO DOMINANCIA INESTABILIDADT0 TANDEMT0 DIANA BY GRUPO

/FORMAT= AVALUE TABLES

/STATISTIC=CHISQ

/CELLS= COUNT

/COUNT ROUND CELL .

ONEWAY

EDAD TACSDR BY GRUPO

/MISSING ANALYSIS

/POSTHOC = BONFERRONI ALPHA(.05).

NPAR TESTS

/K-W= TIEMPOEVOL BY GRUPO(1 3)

/MISSING ANALYSIS.

NPAR TESTS

/K-W=DURACIONQX ENERGIAFINAL POWERFINAL DURACIONFINAL TAVFINAL NSONIC BY

GRUPO(1 3)

/MISSING ANALYSIS.

EXAMINE

VARIABLES=TreatHTT0 TreatHTT1 TreatHTT2 BY GRUPO /COMPARE VARIABLE

/PLOT=BOXPLOT/STATISTICS=NONE/NOTOTAL

/MISSING=PAIRWISE .

GLM

TreatHTT0 TreatHTT1 TreatHTT2 BY GRUPO

/WSFACTOR = tiempo 3 Polynomial

/METHOD = SSTYPE(3)

/SAVE = RESID

/POSTHOC = GRUPO ( BONFERRONI )

/PLOT = PROFILE( tiempo*GRUPO GRUPO*tiempo)

/EMMEANS = TABLES(OVERALL)

/EMMEANS = TABLES(GRUPO)

/EMMEANS = TABLES(GRUPO*tiempo)

/EMMEANS = TABLES(tiempo*GRUPO)

/EMMEANS = TABLES(tiempo) COMPARE ADJ(BONFERRONI)

/EMMEANS = TABLES(tiempo*GRUPO) COMPARE(tiempo)ADJ(BONFERRONI) COMPARE(GRUPO)ADJ(BONFERRONI)

/EMMEANS = TABLES(GRUPO*tiempo) COMPARE(GRUPO)ADJ(BONFERRONI) COMPARE(tiempo)ADJ(BONFERRONI)

/PRINT = HOMOGENEITY

/CRITERIA = ALPHA(.05)

/WSDESIGN = tiempo

/DESIGN = GRUPO .

GLM

TreatHTT0 TreatHTT1 TreatHTT2 BY GRUPO SEXO WITH EDAD

/WSFACTOR = tiempo 3 Polynomial

/METHOD = SSTYPE(3)

/SAVE = RESID

/POSTHOC = GRUPO ( BONFERRONI )

/PLOT = PROFILE( tiempo*GRUPO GRUPO*tiempo)

/EMMEANS = TABLES(OVERALL)

/EMMEANS = TABLES(GRUPO)

/EMMEANS = TABLES(GRUPO*tiempo)

/EMMEANS = TABLES(tiempo*GRUPO)

/EMMEANS = TABLES(tiempo) COMPARE ADJ(BONFERRONI)

/EMMEANS = TABLES(tiempo*GRUPO) COMPARE(tiempo)ADJ(BONFERRONI) COMPARE(GRUPO)ADJ(BONFERRONI)

/EMMEANS = TABLES(GRUPO*tiempo) COMPARE(GRUPO)ADJ(BONFERRONI) COMPARE(tiempo)ADJ(BONFERRONI)

/PRINT = HOMOGENEITY

/CRITERIA = ALPHA(.05)

/WSDESIGN = tiempo

/DESIGN = GRUPO SEXO EDAD .

GRAPH

/SCATTERPLOT(BIVAR)=EDAD WITH TreatHTT0

/MISSING=LISTWISE .

GRAPH

/SCATTERPLOT(BIVAR)=EDAD WITH TreatHTT2

/MISSING=LISTWISE .

EXAMINE

VARIABLES=NoTreatHTT0 NoTreatHTT1 NoTreatHTT2 BY GRUPO /COMPARE VARIABLE

/PLOT=BOXPLOT/STATISTICS=NONE/NOTOTAL

/MISSING=PAIRWISE .

GLM

NoTreatHTT0 NoTreatHTT1 NoTreatHTT2 BY GRUPO

/WSFACTOR = tiempo 3 Polynomial

/METHOD = SSTYPE(3)

/SAVE = RESID

/POSTHOC = GRUPO ( BONFERRONI )

/PLOT = PROFILE( tiempo*GRUPO GRUPO*tiempo)

/EMMEANS = TABLES(OVERALL)

/EMMEANS = TABLES(GRUPO)

/EMMEANS = TABLES(GRUPO*tiempo)

/EMMEANS = TABLES(tiempo*GRUPO)

/EMMEANS = TABLES(tiempo) COMPARE ADJ(BONFERRONI)

/EMMEANS = TABLES(tiempo*GRUPO) COMPARE(tiempo)ADJ(BONFERRONI) COMPARE(GRUPO)ADJ(BONFERRONI)

/EMMEANS = TABLES(GRUPO*tiempo) COMPARE(GRUPO)ADJ(BONFERRONI) COMPARE(tiempo)ADJ(BONFERRONI)

/PRINT = HOMOGENEITY

/CRITERIA = ALPHA(.05)

/WSDESIGN = tiempo

/DESIGN = GRUPO .

GLM

NoTreatHTT0 NoTreatHTT1 NoTreatHTT2 BY GRUPO SEXO WITH EDAD

/WSFACTOR = tiempo 3 Polynomial

/METHOD = SSTYPE(3)

/SAVE = RESID

/POSTHOC = GRUPO ( BONFERRONI )

/PLOT = PROFILE( tiempo*GRUPO GRUPO*tiempo)

/EMMEANS = TABLES(OVERALL)

/EMMEANS = TABLES(GRUPO)

/EMMEANS = TABLES(GRUPO*tiempo)

/EMMEANS = TABLES(tiempo*GRUPO)

/EMMEANS = TABLES(tiempo) COMPARE ADJ(BONFERRONI)

/EMMEANS = TABLES(tiempo*GRUPO) COMPARE(tiempo)ADJ(BONFERRONI) COMPARE(GRUPO)ADJ(BONFERRONI)

/EMMEANS = TABLES(GRUPO*tiempo) COMPARE(GRUPO)ADJ(BONFERRONI) COMPARE(tiempo)ADJ(BONFERRONI)

/PRINT = HOMOGENEITY

/CRITERIA = ALPHA(.05)

/WSDESIGN = tiempo

/DESIGN = GRUPO SEXO EDAD .

GRAPH

/SCATTERPLOT(BIVAR)=EDAD WITH NoTreatHTT0

/MISSING=LISTWISE .

GRAPH

/SCATTERPLOT(BIVAR)=EDAD WITH NoTreatHTT1

/MISSING=LISTWISE .

GRAPH

/SCATTERPLOT(BIVAR)=EDAD WITH NoTreatHTT2

/MISSING=LISTWISE .

*** FTM TOTAL*******.

EXAMINE

VARIABLES=FTMTotalT0 FTMTotalT1 FTMTotalT2 BY GRUPO /COMPARE VARIABLE

/PLOT=BOXPLOT/STATISTICS=NONE/NOTOTAL

/MISSING=PAIRWISE .

GLM

FTMTotalT0 FTMTotalT1 FTMTotalT2 BY GRUPO

/WSFACTOR = tiempo 3 Polynomial

/METHOD = SSTYPE(3)

/SAVE = RESID

/POSTHOC = GRUPO ( BONFERRONI )

/PLOT = PROFILE( tiempo*GRUPO GRUPO*tiempo)

/EMMEANS = TABLES(OVERALL)

/EMMEANS = TABLES(GRUPO)

/EMMEANS = TABLES(GRUPO*tiempo)

/EMMEANS = TABLES(tiempo*GRUPO)

/EMMEANS = TABLES(tiempo) COMPARE ADJ(BONFERRONI)

/EMMEANS = TABLES(tiempo*GRUPO) COMPARE(tiempo)ADJ(BONFERRONI) COMPARE(GRUPO)ADJ(BONFERRONI)

/EMMEANS = TABLES(GRUPO*tiempo) COMPARE(GRUPO)ADJ(BONFERRONI) COMPARE(tiempo)ADJ(BONFERRONI)

/PRINT = HOMOGENEITY

/CRITERIA = ALPHA(.05)

/WSDESIGN = tiempo

/DESIGN = GRUPO .

GLM

FTMTotalT0 FTMTotalT1 FTMTotalT2 BY GRUPO SEXO WITH EDAD

/WSFACTOR = tiempo 3 Polynomial

/METHOD = SSTYPE(3)

/SAVE = RESID

/POSTHOC = GRUPO ( BONFERRONI )

/PLOT = PROFILE( tiempo*GRUPO GRUPO*tiempo)

/EMMEANS = TABLES(OVERALL)

/EMMEANS = TABLES(GRUPO)

/EMMEANS = TABLES(GRUPO*tiempo)

/EMMEANS = TABLES(tiempo*GRUPO)

/EMMEANS = TABLES(tiempo) COMPARE ADJ(BONFERRONI)

/EMMEANS = TABLES(tiempo*GRUPO) COMPARE(tiempo)ADJ(BONFERRONI) COMPARE(GRUPO)ADJ(BONFERRONI)

/EMMEANS = TABLES(GRUPO*tiempo) COMPARE(GRUPO)ADJ(BONFERRONI) COMPARE(tiempo)ADJ(BONFERRONI)

/PRINT = HOMOGENEITY

/CRITERIA = ALPHA(.05)

/WSDESIGN = tiempo

/DESIGN = GRUPO SEXO EDAD .

GRAPH

/SCATTERPLOT(BIVAR)=EDAD WITH FTMTotalT0

/MISSING=LISTWISE .

RECODE EDAD (LOW THRU 65=1)(66 THRU 79=2)(80 THRU HIG=3) INTO EDADRE.

EXECUTE.

RECODE EDAD (LOW THRU 73=1)(74 THRU HIG=2) INTO EDADRE2.

EXECUTE.

EXAMINE

VARIABLES= DISCAPACIDADT0 DISCAPACIDADT1 DISCAPACIDADT2 BY GRUPO /COMPARE VARIABLE

/PLOT=BOXPLOT/STATISTICS=NONE/NOTOTAL

/MISSING=PAIRWISE .

COMPUTE X=(GRUPO=1).

FILTER BY X.

EXECUTE.

COMPUTE X=(GRUPO=2).

FILTER BY X.

EXECUTE.

COMPUTE X=(GRUPO=3).

FILTER BY X.

EXECUTE.

NPAR TEST

/WILCOXON=DISCAPACIDADT0 DISCAPACIDADT1 DISCAPACIDADT0 WITH DISCAPACIDADT1

DISCAPACIDADT2 DISCAPACIDADT2 (PAIRED)

/MISSING ANALYSIS.

FILTER OFF.

EXECUTE.

NPAR TESTS

/M-W= DISCAPACIDADT0 DISCAPACIDADT1 DISCAPACIDADT2 BY GRUPO(1 2)

/M-W= DISCAPACIDADT0 DISCAPACIDADT1 DISCAPACIDADT2 BY GRUPO(1 3)

/M-W= DISCAPACIDADT0 DISCAPACIDADT1 DISCAPACIDADT2 BY GRUPO(2 3)

/MISSING ANALYSIS.

EXAMINE

VARIABLES=MEJORIASUBT1 MEJORIASUBT2 BY GRUPO /COMPARE VARIABLE/PLOT=BOXPLOT

/STATISTICS=NONE/NOTOTAL

/MISSING=LISTWISE .

COMPUTE X=(GRUPO=1).

FILTER BY X.

EXECUTE.

COMPUTE X=(GRUPO=2).

FILTER BY X.

EXECUTE.

COMPUTE X=(GRUPO=3).

FILTER BY X.

EXECUTE.

NPAR TEST

/WILCOXON= MEJORIASUBT1 WITH MEJORIASUBT2 (PAIRED)

/MISSING ANALYSIS.

NPAR TESTS

/M-W= MEJORIASUBT1 MEJORIASUBT2 BY GRUPO(1 2)

/M-W= MEJORIASUBT1 MEJORIASUBT2 BY GRUPO(1 3)

/M-W= MEJORIASUBT1 MEJORIASUBT2 BY GRUPO(2 3)

/MISSING ANALYSIS.

CROSSTABS

/TABLES=DISARTRIAT1 DEBILIDADT1 PARESTESIAST1 DISMETRIAT1 OTROST1

DISARTRIAT2 DEBILIDADT2 PARESTESIAST2 DISMETRIAT2 OTROST2 BY GRUPO

/FORMAT= AVALUE TABLES

/STATISTIC=CHISQ

/CELLS= COUNT

/COUNT ROUND CELL .

COMPUTE X=(GRUPO=1).

FILTER BY X.

EXECUTE.

COMPUTE X=(GRUPO=2).

FILTER BY X.

EXECUTE.

COMPUTE X=(GRUPO=3).

FILTER BY X.

EXECUTE.

CROSSTABS

/TABLES=PARESTESIAST1 BY PARESTESIAST2

/FORMAT= AVALUE TABLES

/STATISTIC=MCNEMAR

/CELLS= COUNT

/COUNT ROUND CELL .

CROSSTABS

/TABLES=DISMETRIAT1 BY DISMETRIAT2

/FORMAT= AVALUE TABLES

/STATISTIC=MCNEMAR

/CELLS= COUNT

/COUNT ROUND CELL .

CROSSTABS

/TABLES=OTROST1 BY OTROST2

/FORMAT= AVALUE TABLES

/STATISTIC=MCNEMAR

/CELLS= COUNT

/COUNT ROUND CELL .

FILTER OFF.

EXECUTE.

COMPUTE X=((GRUPO=1)&(VALIDO=1)).

FILTER BY X.

EXECUTE.

COMPUTE X=((GRUPO=2)&(VALIDO=1)).

FILTER BY X.

EXECUTE.

COMPUTE X=((GRUPO=3)&(VALIDO=1)).

FILTER BY X.

EXECUTE.

CROSSTABS

/TABLES= INESTABILIDADT0 BY INESTABILIDADT1

/TABLES= INESTABILIDADT1 BY INESTABILIDADT2

/TABLES= INESTABILIDADT0 BY INESTABILIDADT2

/FORMAT= AVALUE TABLES

/STATISTIC=MCNEMAR

/CELLS= COUNT

/COUNT ROUND CELL .

FILTER OFF.

EXECUTE.

COMPUTE X=((VALIDO=1)&((GRUPO=1)OR(GRUPO=2))).

FILTER BY X.

EXECUTE.

COMPUTE X=((VALIDO=1)&((GRUPO=1)OR(GRUPO=3))).

FILTER BY X.

EXECUTE.

COMPUTE X=((VALIDO=1)&((GRUPO=2)OR(GRUPO=3))).

FILTER BY X.

EXECUTE.

CROSSTABS

/TABLES=INESTABILIDADT0 INESTABILIDADT1 INESTABILIDADT2 BY GRUPO

/FORMAT= AVALUE TABLES

/STATISTIC=CHISQ

/CELLS= COUNT

/COUNT ROUND CELL .

FILTER OFF.

EXECUTE.

COMPUTE X=((GRUPO=1)&(VALIDO=1)).

FILTER BY X.

EXECUTE.

COMPUTE X=((GRUPO=2)&(VALIDO=1)).

FILTER BY X.

EXECUTE.

COMPUTE X=((GRUPO=3)&(VALIDO=1)).

FILTER BY X.

EXECUTE.

NPAR TEST

/WILCOXON=TANDEMT0 TANDEMT1 TANDEMT0 WITH TANDEMT1

TANDEMT2 TANDEMT2 (PAIRED)

/MISSING ANALYSIS.

FILTER OFF.

EXECUTE.

COMPUTE X=(VALIDO=1).

FILTER BY X.

EXECUTE.

NPAR TESTS

/M-W= TANDEMT0 TANDEMT1 TANDEMT2 BY GRUPO(1 2)

/M-W= TANDEMT0 TANDEMT1 TANDEMT2 BY GRUPO(1 3)

/M-W= TANDEMT0 TANDEMT1 TANDEMT2 BY GRUPO(2 3)

/MISSING ANALYSIS.

FILTER OFF.

EXECUTE.

COMPUTE X=(VALIDO=1).

FILTER BY X.

EXECUTE.

EXAMINE

VARIABLES= BERGT0 BERGT1 BERGT2 BY GRUPO /COMPARE VARIABLE

/PLOT=BOXPLOT/STATISTICS=NONE/NOTOTAL

/MISSING=PAIRWISE .

COMPUTE X=((GRUPO=1)&(VALIDO=1)).

FILTER BY X.

EXECUTE.

COMPUTE X=((GRUPO=2)&(VALIDO=1)).

FILTER BY X.

EXECUTE.

COMPUTE X=((GRUPO=3)&(VALIDO=1)).

FILTER BY X.

EXECUTE.

NPAR TEST

/WILCOXON=BERGT0 BERGT1 BERGT0 WITH BERGT1

BERGT2 BERGT2 (PAIRED)

/MISSING ANALYSIS.

FILTER OFF.

EXECUTE.

COMPUTE X=(VALIDO=1).

FILTER BY X.

EXECUTE.

NPAR TESTS

/M-W= BERGT0 BERGT1 BERGT2 BY GRUPO(1 2)

/M-W= BERGT0 BERGT1 BERGT2 BY GRUPO(1 3)

/M-W= BERGT0 BERGT1 BERGT2 BY GRUPO(2 3)

/MISSING ANALYSIS.

FILTER OFF.

EXECUTE.

EXAMINE

VARIABLES=TreatHTT0 TreatHTT1 TreatHTT2 BY TANDEMT0RE /COMPARE VARIABLE

/PLOT=BOXPLOT/STATISTICS=NONE/NOTOTAL

/MISSING=PAIRWISE .

GLM

TreatHTT0 TreatHTT1 TreatHTT2 BY TANDEMT0RE

/WSFACTOR = tiempo 3 Polynomial

/METHOD = SSTYPE(3)

/SAVE = RESID

/POSTHOC = TANDEMT0RE ( BONFERRONI )

/PLOT = PROFILE( tiempo*TANDEMT0RE TANDEMT0RE*tiempo)

/EMMEANS = TABLES(OVERALL)

/EMMEANS = TABLES(TANDEMT0RE)

/EMMEANS = TABLES(TANDEMT0RE*tiempo)

/EMMEANS = TABLES(tiempo*TANDEMT0RE)

/EMMEANS = TABLES(tiempo) COMPARE ADJ(BONFERRONI)

/EMMEANS = TABLES(tiempo*TANDEMT0RE) COMPARE(tiempo)ADJ(BONFERRONI) COMPARE(TANDEMT0RE)ADJ(BONFERRONI)

/EMMEANS = TABLES(TANDEMT0RE*tiempo) COMPARE(TANDEMT0RE)ADJ(BONFERRONI) COMPARE(tiempo)ADJ(BONFERRONI)

/PRINT = HOMOGENEITY

/CRITERIA = ALPHA(.05)

/WSDESIGN = tiempo

/DESIGN = TANDEMT0RE .

GLM

TreatHTT0 TreatHTT1 TreatHTT2 BY TANDEMT0RE SEXO WITH EDAD

/WSFACTOR = tiempo 3 Polynomial

/METHOD = SSTYPE(3)

/SAVE = RESID

/POSTHOC = TANDEMT0RE ( BONFERRONI )

/PLOT = PROFILE( tiempo*TANDEMT0RE TANDEMT0RE*tiempo)

/EMMEANS = TABLES(OVERALL)

/EMMEANS = TABLES(TANDEMT0RE)

/EMMEANS = TABLES(TANDEMT0RE*tiempo)

/EMMEANS = TABLES(tiempo*TANDEMT0RE)

/EMMEANS = TABLES(tiempo) COMPARE ADJ(BONFERRONI)

/EMMEANS = TABLES(tiempo*TANDEMT0RE) COMPARE(tiempo)ADJ(BONFERRONI) COMPARE(TANDEMT0RE)ADJ(BONFERRONI)

/EMMEANS = TABLES(TANDEMT0RE*tiempo) COMPARE(TANDEMT0RE)ADJ(BONFERRONI) COMPARE(tiempo)ADJ(BONFERRONI)

/PRINT = HOMOGENEITY

/CRITERIA = ALPHA(.05)

/WSDESIGN = tiempo

/DESIGN = TANDEMT0RE SEXO EDAD .

GRAPH

/SCATTERPLOT(BIVAR)=EDAD WITH TreatHTT0

/MISSING=LISTWISE .

GRAPH

/SCATTERPLOT(BIVAR)=EDAD WITH TreatHTT2

/MISSING=LISTWISE .

EXAMINE

VARIABLES=NoTreatHTT0 NoTreatHTT1 NoTreatHTT2 BY TANDEMT0RE /COMPARE VARIABLE

/PLOT=BOXPLOT/STATISTICS=NONE/NOTOTAL

/MISSING=PAIRWISE .

GLM

NoTreatHTT0 NoTreatHTT1 NoTreatHTT2 BY TANDEMT0RE

/WSFACTOR = tiempo 3 Polynomial

/METHOD = SSTYPE(3)

/SAVE = RESID

/POSTHOC = TANDEMT0RE ( BONFERRONI )

/PLOT = PROFILE( tiempo*TANDEMT0RE TANDEMT0RE*tiempo)

/EMMEANS = TABLES(OVERALL)

/EMMEANS = TABLES(TANDEMT0RE)

/EMMEANS = TABLES(TANDEMT0RE*tiempo)

/EMMEANS = TABLES(tiempo*TANDEMT0RE)

/EMMEANS = TABLES(tiempo) COMPARE ADJ(BONFERRONI)

/EMMEANS = TABLES(tiempo*TANDEMT0RE) COMPARE(tiempo)ADJ(BONFERRONI) COMPARE(TANDEMT0RE)ADJ(BONFERRONI)

/EMMEANS = TABLES(TANDEMT0RE*tiempo) COMPARE(TANDEMT0RE)ADJ(BONFERRONI) COMPARE(tiempo)ADJ(BONFERRONI)

/PRINT = HOMOGENEITY

/CRITERIA = ALPHA(.05)

/WSDESIGN = tiempo

/DESIGN = TANDEMT0RE .

GLM

NoTreatHTT0 NoTreatHTT1 NoTreatHTT2 BY TANDEMT0RE SEXO WITH EDAD

/WSFACTOR = tiempo 3 Polynomial

/METHOD = SSTYPE(3)

/SAVE = RESID

/POSTHOC = TANDEMT0RE ( BONFERRONI )

/PLOT = PROFILE( tiempo*TANDEMT0RE TANDEMT0RE*tiempo)

/EMMEANS = TABLES(OVERALL)

/EMMEANS = TABLES(TANDEMT0RE)

/EMMEANS = TABLES(TANDEMT0RE*tiempo)

/EMMEANS = TABLES(tiempo*TANDEMT0RE)

/EMMEANS = TABLES(tiempo) COMPARE ADJ(BONFERRONI)

/EMMEANS = TABLES(tiempo*TANDEMT0RE) COMPARE(tiempo)ADJ(BONFERRONI) COMPARE(TANDEMT0RE)ADJ(BONFERRONI)

/EMMEANS = TABLES(TANDEMT0RE*tiempo) COMPARE(TANDEMT0RE)ADJ(BONFERRONI) COMPARE(tiempo)ADJ(BONFERRONI)

/PRINT = HOMOGENEITY

/CRITERIA = ALPHA(.05)

/WSDESIGN = tiempo

/DESIGN = TANDEMT0RE SEXO EDAD .

GRAPH

/SCATTERPLOT(BIVAR)=EDAD WITH NoTreatHTT0

/MISSING=LISTWISE .

GRAPH

/SCATTERPLOT(BIVAR)=EDAD WITH NoTreatHTT1

/MISSING=LISTWISE .

GRAPH

/SCATTERPLOT(BIVAR)=EDAD WITH NoTreatHTT2

/MISSING=LISTWISE .

EXAMINE

VARIABLES=FTMTotalT0 FTMTotalT1 FTMTotalT2 BY TANDEMT0RE /COMPARE VARIABLE

/PLOT=BOXPLOT/STATISTICS=NONE/NOTOTAL

/MISSING=PAIRWISE .

GLM

FTMTotalT0 FTMTotalT1 FTMTotalT2 BY TANDEMT0RE

/WSFACTOR = tiempo 3 Polynomial

/METHOD = SSTYPE(3)

/SAVE = RESID

/POSTHOC = TANDEMT0RE ( BONFERRONI )

/PLOT = PROFILE( tiempo*TANDEMT0RE TANDEMT0RE*tiempo)

/EMMEANS = TABLES(OVERALL)

/EMMEANS = TABLES(TANDEMT0RE)

/EMMEANS = TABLES(TANDEMT0RE*tiempo)

/EMMEANS = TABLES(tiempo*TANDEMT0RE)

/EMMEANS = TABLES(tiempo) COMPARE ADJ(BONFERRONI)

/EMMEANS = TABLES(tiempo*TANDEMT0RE) COMPARE(tiempo)ADJ(BONFERRONI) COMPARE(TANDEMT0RE)ADJ(BONFERRONI)

/EMMEANS = TABLES(TANDEMT0RE*tiempo) COMPARE(TANDEMT0RE)ADJ(BONFERRONI) COMPARE(tiempo)ADJ(BONFERRONI)

/PRINT = HOMOGENEITY

/CRITERIA = ALPHA(.05)

/WSDESIGN = tiempo

/DESIGN = TANDEMT0RE .

GLM

FTMTotalT0 FTMTotalT1 FTMTotalT2 BY TANDEMT0RE SEXO WITH EDAD

/WSFACTOR = tiempo 3 Polynomial

/METHOD = SSTYPE(3)

/SAVE = RESID

/POSTHOC = TANDEMT0RE ( BONFERRONI )

/PLOT = PROFILE( tiempo*TANDEMT0RE TANDEMT0RE*tiempo)

/EMMEANS = TABLES(OVERALL)

/EMMEANS = TABLES(TANDEMT0RE)

/EMMEANS = TABLES(TANDEMT0RE*tiempo)

/EMMEANS = TABLES(tiempo*TANDEMT0RE)

/EMMEANS = TABLES(tiempo) COMPARE ADJ(BONFERRONI)

/EMMEANS = TABLES(tiempo*TANDEMT0RE) COMPARE(tiempo)ADJ(BONFERRONI) COMPARE(TANDEMT0RE)ADJ(BONFERRONI)

/EMMEANS = TABLES(TANDEMT0RE*tiempo) COMPARE(TANDEMT0RE)ADJ(BONFERRONI) COMPARE(tiempo)ADJ(BONFERRONI)

/PRINT = HOMOGENEITY

/CRITERIA = ALPHA(.05)

/WSDESIGN = tiempo

/DESIGN = TANDEMT0RE SEXO EDAD .

GRAPH

/SCATTERPLOT(BIVAR)=EDAD WITH FTMTotalT0

/MISSING=LISTWISE .

EXAMINE

VARIABLES= DISCAPACIDADT0 DISCAPACIDADT1 DISCAPACIDADT2 BY TANDEMT0RE /COMPARE VARIABLE

/PLOT=BOXPLOT/STATISTICS=NONE/NOTOTAL

/MISSING=PAIRWISE .

COMPUTE X=(TANDEMT0RE=0).

FILTER BY X.

EXECUTE.

COMPUTE X=(TANDEMT0RE=1).

FILTER BY X.

EXECUTE.

NPAR TEST

/WILCOXON=DISCAPACIDADT0 DISCAPACIDADT1 DISCAPACIDADT0 WITH DISCAPACIDADT1

DISCAPACIDADT2 DISCAPACIDADT2 (PAIRED)

/MISSING ANALYSIS.

FILTER OFF.

EXECUTE.

NPAR TESTS

/M-W= DISCAPACIDADT0 DISCAPACIDADT1 DISCAPACIDADT2 BY TANDEMT0RE(0 1)

/MISSING ANALYSIS.

EXAMINE

VARIABLES=MEJORIASUBT1 MEJORIASUBT2 BY TANDEMT0RE /COMPARE VARIABLE/PLOT=BOXPLOT

/STATISTICS=NONE/NOTOTAL

/MISSING=LISTWISE .

COMPUTE X=(TANDEMT0RE=0).

FILTER BY X.

EXECUTE.

COMPUTE X=(TANDEMT0RE=1).

FILTER BY X.

EXECUTE.

NPAR TEST

/WILCOXON= MEJORIASUBT1 WITH MEJORIASUBT2 (PAIRED)

/MISSING ANALYSIS.

FILTER OFF.

EXECUTE.

NPAR TESTS

/M-W= MEJORIASUBT1 MEJORIASUBT2 BY TANDEMT0RE(0 1)

/MISSING ANALYSIS.

*****************************************************.

***** code for analysis in R **********.

*****************************************************.

datos<-read.delim("clipboard")

datos

attach(datos)

res<-f1.ld.f1(y=DISCAP, time=TIEMPO, group=GRUPO, subject=ID, time.name="Tiempo", group.name="Grupo")

res$ANOVA.test

res<-f2.ld.f1(y=DISCAP, time=TIEMPO, group1=EDADRE2, group2=GRUPO, subject=ID, time.name="Tiempo", group1.name="Edad", group2.name="Grupo")

res$ANOVA.test

res<-f1.ld.f1(y=INESTAB, time=TIEMPO, group=GRUPO, subject=ID, time.name="Tiempo", group.name="Grupo")

res$ANOVA.test

res<-ld.f1(y=INESTAB, time=TIEMPO, subject=ID, time.name="Tiempo")

res$ANOVA.test

res<-f2.ld.f1(y=INESTAB, time=TIEMPO, group1=EDADRE2, group2=GRUPO, subject=ID, time.name="Tiempo", group1.name="Edad", group2.name="Grupo")

res$ANOVA.test

res<-f1.ld.f1(y=TANDEM, time=TIEMPO, group=GRUPO, subject=ID, time.name="Tiempo", group.name="Grupo")

res$ANOVA.test

res<-f2.ld.f1(y=TANDEM, time=TIEMPO, group1=EDADRE2, group2=GRUPO, subject=ID, time.name="Tiempo", group1.name="Edad", group2.name="Grupo")

res$ANOVA.test

res<-f2.ld.f1(y=TANDEM, time=TIEMPO, group1=EDADRE2, group2=GRUPO, subject=ID, time.name="Tiempo", group1.name="Edad", group2.name="Grupo")

res$ANOVA.test

res<-f1.ld.f1(y=BERG, time=TIEMPO, group=GRUPO, subject=ID, time.name="Tiempo", group.name="Grupo")

res$ANOVA.test

res<-f2.ld.f1(y=BERG, time=TIEMPO, group1=EDADRE2, group2=GRUPO, subject=ID, time.name="Tiempo", group1.name="Edad", group2.name="Grupo")

res$ANOVA.test

res<-f2.ld.f1(y=BERG, time=TIEMPO, group1=EDADRE2, group2=GRUPO, subject=ID, time.name="Tiempo", group1.name="Edad", group2.name="Grupo")

res$ANOVA.test

res<-f2.ld.f1(y=BERG, time=TIEMPO, group1=EDADRE2, group2=GRUPO, subject=ID, time.name="Tiempo", group1.name="Edad", group2.name="Grupo")

res$ANOVA.test

datos<-read.delim("clipboard")

datos

attach(datos)

res<-f1.ld.f1(y=INESTAB, time=TIEMPO, group=TANDEMT0RE, subject=ID, time.name="Tiempo", group.name="Tándem T0 ")

res$ANOVA.test

res<-ld.f1(y=INESTAB, time=TIEMPO, subject=ID, time.name="Tiempo")

res$ANOVA.test

res<-f2.ld.f1(y=INESTAB, time=TIEMPO, group1=EDADRE, group2=TANDEMT0RE, subject=ID, time.name="Tiempo", group1.name="Edad", group2.name="Tándem T0 ")

res$ANOVA.test

res<-f2.ld.f1(y=INESTAB, time=TIEMPO, group1=EDADRE2, group2=TANDEMT0RE, subject=ID, time.name="Tiempo", group1.name="Edad", group2.name="Tándem T0 ")

res$ANOVA.test

res<-f1.ld.f1(y=TANDEM, time=TIEMPO, group=TANDEMT0RE, subject=ID, time.name="Tiempo", group.name="Tándem T0 ")

res$ANOVA.test

res<-f2.ld.f1(y=TANDEM, time=TIEMPO, group1=EDADRE2, group2=TANDEMT0RE, subject=ID, time.name="Tiempo", group1.name="Edad", group2.name="Tándem T0 ")

res$ANOVA.test

res<-f2.ld.f1(y=TANDEM, time=TIEMPO, group1=EDADRE2, group2=TANDEMT0RE, subject=ID, time.name="Tiempo", group1.name="Edad", group2.name="Tándem T0 ")

res$ANOVA.test

res<-f1.ld.f1(y=BERG, time=TIEMPO, group=TANDEMT0RE, subject=ID, time.name="Tiempo", group.name="Tándem T0 ")

res$ANOVA.test

res<-f2.ld.f1(y=BERG, time=TIEMPO, group1=EDADRE2, group2=TANDEMT0RE, subject=ID, time.name="Tiempo", group1.name="Edad", group2.name="Tándem T0 ")

res$ANOVA.test

res<-f2.ld.f1(y=BERG, time=TIEMPO, group1=EDADRE2, group2=TANDEMT0RE, subject=ID, time.name="Tiempo", group1.name="Edad", group2.name="Tándem T0 ")

res$ANOVA.test

res<-f2.ld.f1(y=BERG, time=TIEMPO, group1=EDADRE2, group2=TANDEMT0RE, subject=ID, time.name="Tiempo", group1.name="Edad", group2.name="Tándem T0 ")

res$ANOVA.test

res<-f1.ld.f1(y=DISCAP, time=TIEMPO, group=TANDEMT0RE, subject=ID, time.name="Tiempo", group.name="Tándem T0 ")

res$ANOVA.test

res<-f2.ld.f1(y=DISCAP, time=TIEMPO, group1=EDADRE2, group2=TANDEMT0RE, subject=ID, time.name="Tiempo", group1.name="Edad", group2.name="Tándem T0 ")

res$ANOVA.test
